# Quail Egg-Based Supplements in Allergic Rhinitis: A Systematic Review of Clinical Studies

**DOI:** 10.3390/nu17040712

**Published:** 2025-02-17

**Authors:** Michele Antonelli, Elena Mazzoleni, Davide Donelli

**Affiliations:** 1 Private Practice for Evidence-Based Integrative and Preventive Medicine, 42025 Cavriago, Italy; 2 Department of Biomedical, Metabolic and Neural Sciences, Università di Modena e Reggio Emilia—UNIMORE, 41121 Modena, Italy; 3 Cardiology Unit, Azienda Ospedaliero-Universitaria di Parma—AOUP, 43126 Parma, Italy

**Keywords:** allergy, dietary supplement, quail egg, review, integrative medicine

## Abstract

**Background/Objectives**: This systematic review evaluates the efficacy of quail egg-based supplements (QES) as an integrative remedy for treating allergic rhinitis. **Methods**: A comprehensive search of PubMed, Scopus, EMBASE, Cochrane Library, and Google Scholar was conducted up to January 2025 to address the research question. **Results**: A total of 294 studies were initially identified, with five clinical reports meeting the inclusion criteria. Participant numbers ranged from 40 to 180 (median: 77), with a balanced gender ratio. Four reports focused on allergic rhinitis, and one investigated nonsymptomatic atopic individuals exposed to volatile allergens. The findings suggest that a combination of QES and zinc significantly improves peak nasal inspiratory flow, mucociliary transport time, and symptoms such as rhinorrhea, nasal congestion, itchy nose and eyes, and sneezing in patients with allergic rhinitis. Additionally, QES may reduce the reliance on standard symptomatic medications. The intervention was generally well tolerated, with side effects being rare, mild, and transient; however, QES should be avoided in patients with egg allergies. **Conclusions**: The reviewed studies indicate that QES with zinc can serve as an effective integrative approach to alleviating symptoms of allergic rhinitis. Further research is recommended to confirm these findings.

## 1. Introduction

Seasonal respiratory allergies, most commonly manifesting as allergic rhinitis, affect millions of individuals worldwide, causing a range of symptoms that can significantly diminish these individual’s quality of life [1]. Allergic rhinitis occurs when the immune system reacts excessively to airborne allergens such as pollen, mold spores, dust mites, or animal dander [2]. This overreaction triggers the release of histamines and other inflammatory mediators, leading to typical symptoms such as sneezing, nasal congestion, itchy eyes, and a runny nose [3]. While these symptoms are generally more prevalent during certain seasons, such as spring, when pollen levels are highest, the condition can persist year-round in individuals exposed to perennial allergens [4]. Chronic inflammation associated with allergic rhinitis can also contribute to more severe health complications, such as sinus infections, sleep disturbances, and asthma, highlighting the importance of effective management strategies [5,6].

Traditional treatment options for allergic rhinitis primarily include antihistamines, corticosteroids, and immunotherapy [7]. In particular, immunotherapy works by shifting immunoglobulin (Ig) production from allergen-specific IgE to IgG, with administration routes varying by allergy type: injectable for hymenoptera stings, oral for food allergies, and sublingual or injectable for respiratory allergies, often using modified allergens or allergenic components [8,9,10]. On the contrary, cortisone works by reducing inflammation and suppressing the immune response through the inhibition of pro-inflammatory cytokines, while antihistamines block histamine receptors, preventing the effects of histamine release, such as swelling, itching, and allergic reactions [11,12,13,14]. However, many individuals seek integrative therapies because of concerns over side effects or a desire for more natural approaches to treatment: as a result, there is increasing interest in the role of nutraceuticals, botanicals, and dietary supplementation in managing allergic conditions [15,16,17]. Among the various natural products gaining attention, there are quail (*Coturnix*) egg-based supplements (QES): in particular, quail eggs have long been valued for their nutritional profile, containing high levels of proteins and micronutrients [18] (see Table 1 for further details), and recent studies suggest that they possess potential anti-inflammatory and immune-modulating properties, which may help in regulating the body’s immune response to allergens [19]. Early evidence indicates that quail eggs could potentially mitigate the symptoms of seasonal allergies by reducing inflammatory reactions in the respiratory system, thereby offering an integrative approach to conventional allergy treatments [20].

This research will focus on investigating the potential therapeutic effects and clinical tolerability of QES in managing allergic rhinitis.

## 2. Methods

This research was designed as a systematic review of the scientific literature to evaluate the effects of dietary supplements derived from quail eggs on allergic diseases. The PRISMA guidelines were followed [22], and the review protocol was published in searchRxiv—CABI Digital Library under the following DOI: https://doi.org/10.1079/searchRxiv.2025.00842. A comprehensive search was conducted in January 2025 across multiple electronic databases, including PubMed, Scopus, EMBASE, Cochrane Library, and Google Scholar.

The search strategy for PubMed was as follows: (quail[Title/Abstract] OR Coturnix[Title/Abstract]) AND egg[Title/Abstract] AND (allergic[Title/Abstract] OR allergy[Title/Abstract] OR rhinitis[Title/Abstract] OR rhinosinusitis[Title/Abstract] OR nasal[Title/Abstract] OR IgE[Title/Abstract] OR eosinophil*[Title/Abstract] OR season*[Title/Abstract] OR hypersensitivity[Title/Abstract]). Similar search terms and strategies were employed in other databases to capture relevant studies related to quail egg consumption and allergic diseases, for example:

Scopus: (quail OR Coturnix) AND (egg) AND (allergic OR allergy OR rhinitis OR rhinosinusitis OR nasal OR IgE OR eosinophil* OR season* OR hypersensitivity) (keywords were searched in article abstracts).

EMBASE: (‘quail’:ab,ti OR ‘Coturnix’:ab,ti) AND ‘egg’:ab,ti AND (‘allergic’:ab,ti OR ‘allergy’:ab,ti OR ‘rhinitis’:ab,ti OR ‘rhinosinusitis’:ab,ti OR ‘nasal’:ab,ti OR ‘IgE’:ab,ti OR ‘eosinophil*’:ab,ti OR ‘season*’ OR ‘hypersensitivity’:ab,ti).

Cochrane Library: Trials matching “quail egg” in Title Abstract Keyword—(Word variations have been searched).

Google Scholar: “quail egg” AND “allergy” AND “trial”.

Studies were selected according to the following inclusion criteria based on the PICOS framework:

- P (Population): Atopic subjects with hypersensitivity to respiratory allergens and patients diagnosed with allergic rhinitis based on international clinical standards.
- I (Intervention): Quail egg-based dietary supplements, either alone or in combination with other nutraceuticals (e.g., vitamins and minerals).
- C (Comparator): Any type of comparator, including placebo, other treatments, or no control group.
- O (Outcomes): Clinical outcomes such as changes in disease symptoms, laboratory test results, radiological findings, and health-related quality of life.
- S (Study Design): Clinical studies with a focus on randomized controlled trials (RCTs). Other human studies, such as quasi-experimental, pre-post, and observational studies, were also included to provide a comprehensive overview of the evidence.

Two authors (M.A. and E.M.) independently screened the titles, abstracts, and full texts of the identified articles for eligibility. Disagreements regarding inclusion were resolved through discussion with a third author (D.D.). There were no language restrictions imposed on the search to maximize comprehensiveness. However, only studies published in scientific journals were included to ensure the evidence was peer-reviewed.

The quality of the included studies was assessed using the National Institutes of Health (NIH) quality assessment tools, available at https://www.nhlbi.nih.gov/health-topics/study-quality-assessment-tools, accessed on 23 January 2025. This evaluation was carried out independently by two authors (M.A. and E.M.), and any discrepancies were addressed by a third author (D.D.). In particular, the quality of the included studies was assessed using two standardized tools: the Quality Assessment of Controlled Intervention Studies and the Quality Assessment Tool for Before–After (Pre-Post) Studies With No Control Group. These tools evaluate key methodological criteria such as randomization, blinding, baseline comparability, adherence to intervention protocols, outcome measurement reliability, sample size adequacy, and statistical analysis.

Due to the limited and heterogeneous nature of available data, no quantitative synthesis was performed. Therefore, the findings from the included studies were presented qualitatively.

## 3. Results

**Figure 1:**
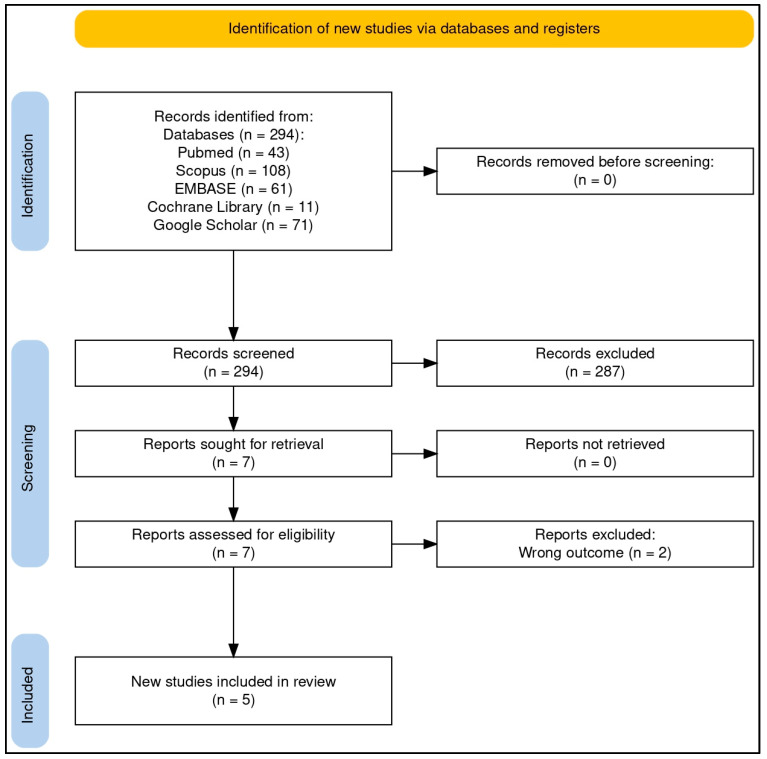
PRISMA flow diagram of the article selection process. The flow diagram was created online with the help of a dedicated tool (“PRISMA2020”, an Open Source R package and web-based Shiny app—version: 06.2022) [30].

The literature search identified 294 results, from which five reports were included in this review (refer to Figure 1 for a brief overview of the article selection process): one report summarizing five RCTs [23], two additional RCTs each described in separate reports [24,25], and two pre-post studies presented in individual reports [26,27]. These studies investigated the effects of QES combined with zinc in the treatment of allergic conditions of the upper airways.

Of the 294 research items retrieved, approximately 57% (n = 168) were duplicates. The remaining studies underwent title and abstract screening, with the majority excluded for being outside the scope of this review. Overall, 52% were original research, including clinical studies and laboratory experiments, 20% focused on quail biology in veterinary science, 16% were case reports on allergic reactions to egg consumption, 8% consisted of conference papers, commentaries, or research letters, and 4% were review articles. Ultimately, seven papers were selected for full-text screening, of which two were excluded as they were surveys on the use of complementary therapies in allergic disorders [28,29] (see Figure 1 for additional details).

Table 2 summarizes the population characteristics, interventions, outcomes, study designs, and key findings for each of the included studies, providing evidence that QES combined with zinc may be an effective treatment for improving respiratory function and alleviating symptoms in patients with allergic rhinitis.

The findings from these studies suggest that QES with zinc can lead to improvements in various outcomes, including peak nasal inspiratory flow (PNIF), mucociliary transport time (MTT), and symptoms such as rhinorrhea, nasal congestion, itchy nose and eyes, and sneezing (see Table 2 for additional details). The studies utilized a variety of different outcome measures, such as the Total Nasal Symptom Score (TNSS), the Sino-Nasal Outcome Test (SNOT-20), and the Rhinitis Control Assessment Test (RCAT), which collectively demonstrated significant improvements in the intervention groups. Overall, no significant differences in IgE levels were observed between the intervention and control groups.

For instance, in the first study, five RCTs collectively involving 690 allergic subjects were summarized (one involving asthmatic patients and the remaining focused on individuals of all ages with allergic rhinitis): clinical evaluations revealed a significant symptomatic improvement and reduction in medication use among patients treated with the supplement compared with those receiving placebo tablets [23]. In another study, 43 nonsymptomatic atopic subjects exposed to an allergenic challenge showed significant improvements in PNIF and visual analog scale (VAS) scores for nasal obstruction after taking QES combined with zinc, compared with the placebo [24]. Another study of 40 patients with mild-to-severe allergic rhinitis demonstrated a significant reduction in TNSS and RCAT scores with QES and zinc in conjunction with mometasone nasal spray, though there was no significant difference in IgE levels between the groups [25]. Additionally, in a pre-post study involving 77 patients, QES plus zinc significantly improved PNIF and VAS scores for rhinorrhea within one day of use, with continued improvements after one week [26]. Last, a study of 45 patients with moderate-to-severe allergic rhinitis showed improvements in SNOT-20 scores and mucociliary transport time after 30 days of treatment with QES and zinc [27].

Most studies in this review focused on patients with allergic rhinitis, while one study involved nonsymptomatic atopic individuals who underwent an allergic challenge before receiving the supplement [24]. The number of participants per study ranged from 40 to 180, with a median of 77 (see Table 2). The intervention consisted of QES combined with zinc, either as a standalone treatment or as an adjunct to standard antiallergic pharmacotherapy, and three proprietary supplements were evaluated: SniZtop^®^, Narivent^®^, and Ovix^®^ (see Table 2 for details). Specifically, each tablet of SniZtop^®^ and Narivent^®^ contained 42 mg of quail egg homogenate and 0.75 mg of zinc, while each Ovix^®^ tablet contained 30 mg of quail egg homogenate and 0.53 mg of zinc. The daily dosage ranged from one to six tablets, depending on symptom severity and the physician’s assessment; the treatment duration ranged from a few days to over a year, with an average duration of approximately one month (Table 2).

The quality assessment of the included studies is summarized in Table 3. Some limitations were identified in the reporting of these studies: specifically, there is insufficient information regarding the blinding procedures for outcome assessors, which raises concerns about potential biases in the assessment of results. Additionally, many studies fail to disclose the methodology used to determine whether the sample size was adequately powered to detect statistically significant outcomes. This lack of information in sample size calculations makes it difficult to assess the generalizability of the findings across the studies. However, while there were some areas for improvement, the majority of the studies demonstrated quite sound methodology, supporting the overall reliability of their findings.

## 4. Discussion

### 4.1. Critical Overview of the Available Evidence

**Figure 2:**
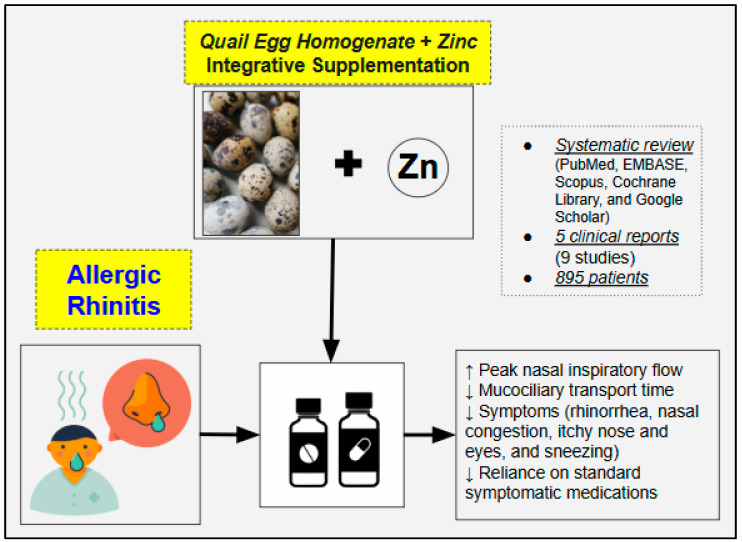
Summary of the findings from the included studies investigating the potential therapeutic effects of QES in the management of allergic rhinitis.

The review findings suggest that QES combined with zinc has the potential to alleviate allergic rhinitis symptoms (Figure 2) and may reduce the reliance on standard symptomatic medications, thereby potentially minimizing the risk of adverse effects associated with conventional pharmacotherapy. Specifically, in mild disease forms or individuals with simply an allergic predisposition, QES plus zinc may help mitigate symptom onset and severity, while in moderate-to-severe cases, QES can serve as a complementary addition to standard pharmacotherapy. The average daily supplemental dose of quail egg homogenate is approximately 84 mg, with higher doses administered in cases of more severe symptoms. In general, by effectively improving allergic rhinitis, synergistic and well-integrated treatment regimes can have the potential to disrupt the progression from seasonal to chronic rhinitis and, ultimately, to asthma [6], thereby slowing the trajectory of the so-called “Atopic March”. This concept highlights the interconnected nature of allergic diseases, where early and targeted intervention in allergic rhinitis may help reduce inflammation, improve airway function, and prevent the escalation of allergic disorders over time [31,32]. This is especially crucial for certain forms of allergic rhinitis, including those characterized by polysensitization and those involving sensitization to house dust mites regardless of other allergens, as they are associated with a heightened risk of developing asthma [33].

Quail eggs are an excellent source of essential micronutrients, including iron, zinc, selenium, and vitamins A, D, and E, as well as beneficial fatty acids (Table 1). These nutrients play a critical role in supporting the immune system and managing allergic responses [34]. For individuals with allergies, deficiencies in these key micronutrients are often linked to poorer clinical outcomes, as they are essential for reducing inflammation, maintaining mucosal barrier integrity, and modulating immune activity [35]. All supplements analyzed in the included studies contained both quail egg homogenate and zinc (Table 2). While the role of zinc in allergic disorders is not yet fully understood, evidence suggests that this micronutrient plays a beneficial role in immune regulation and inflammatory responses: in particular, zinc deficiency, affecting over two billion people worldwide [36], has been linked to more severe allergic manifestations, and its supplementation may help mitigate these effects [37]. Additionally, zinc bioavailability decreases under inflammatory conditions, and its levels tend to be lower in atopic individuals, likely due to persistent low-grade inflammation [38].

Laboratory studies demonstrate that quail egg possesses anti-allergic and anti-inflammatory properties, with its albumen acting as a mast cell stabilizer: in a passive cutaneous anaphylaxis mouse model, quail egg administration significantly reduced mast cell degranulation and vascular permeability [39]. In vitro experiments using human mast cells (HMC-1) demonstrated that quail egg albumen effectively suppressed the release of allergy-related mediators such as β-hexosaminidase, histamine, tryptase, and pro-inflammatory cytokines while also upregulating IL-10: mechanistically, quail egg albumen down-regulated calcium-related proteins (TRPC1, Orai1, STIM1, PLC-γ, and IP3R) and inhibited key signaling pathways (PAR-2, JNK, IKKα, p50, and p65), suggesting its potential in modulating allergic responses [39]. Although quail egg yolk also exhibited immunomodulatory effects by suppressing type 2 cytokines at higher concentrations, the findings highlight the complementary roles of quail egg albumen and yolk in mitigating allergic reactions, supporting its potential as a natural anti-allergic nutrient. Additionally, daily oral administration of quail egg, a known serine protease inhibitor, alleviated symptoms and immune responses by reducing eosinophilic inflammation in mice models [19]. In the same experiment, the treatment also managed to decrease serum levels of tryptase, eosinophil cationic protein, antigen-specific IgE and IgG1, and inflammatory gene expression while increasing IL-10 levels; furthermore, quail egg inhibited PAR-2 activation and NF-κB p65 signaling in inflamed tissues, suggesting its potential as a therapeutic agent for allergic conditions through immune modulation and inflammation control [19]. In summary, based on the available evidence, QES likely exerts its therapeutic effects on allergic reactions by reducing mast cell activation, suppressing type 2 cytokines, attenuating eosinophilic responses, enhancing IL-10 levels, and alleviating allergy-induced inflammation. However, it does not appear to significantly modulate IgE levels, as also reported in the included studies (Table 2). This distinction is important for optimizing treatment strategies, as QES supplementation can be integrated with other medications that target different mechanisms, allowing for a more synergistic approach to managing allergic rhinitis.

Emerging evidence suggests that early-life nutrition plays a crucial role in shaping immune responses and potentially preventing allergic disorders through epigenetic mechanisms, such as DNA methylation, histone modifications, and microRNA regulation [40]. Given that bioactive nutritional components can influence immune tolerance and allergic susceptibility, QES may hold promise in this context: quail eggs contain immunomodulatory compounds that can regulate mast cell activity and inflammation, potentially contributing to a more balanced immune response. While current studies focus on QES for symptom relief in allergic rhinitis, future research should explore their role in early-life nutrition and immune programming. Investigating whether QES supplementation during critical developmental windows, such as childhood, can positively influence epigenetic patterns and reduce the risk of allergic diseases could open new avenues for preventive strategies.

QES for allergic rhinitis appears to be well-tolerated, as side effects reported in the included studies were rare, mild, and transient (see Table 4 for more details). However, caution is necessary when considering QES for patients with egg allergies. In particular, a reported case described a patient who experienced an anaphylactic reaction to raw quail egg while tolerating both cooked quail egg and chicken egg without symptoms [41]. Diagnostic evaluations identified ovotransferrin as the allergenic protein in quail egg white, with no cross-reactivity to chicken egg proteins. Other studies suggest that patients with a history of chicken egg allergy who have subsequently developed tolerance may not need to avoid quail eggs [42]. However, individual screening is essential for safety, as reports suggest potential cross-reactivity between chicken and quail eggs, with ten identified allergens in chicken eggs (ovalbumin being the most abundant protein and ovomucoid the primary allergen in heated egg allergies), both of which are also found in quail eggs, though with qualitative and quantitative variations [43]. In general, an egg allergy typically manifests in early childhood and may resolve with age (importantly, chicken egg allergies do not usually develop in adulthood, apart from exceptional cases [44]) [45,46]. In brief, while QES can be a promising adjunct for managing allergic rhinitis, it is important to assess egg allergies before use: in cases of anamnestic uncertainty, measuring specific IgE levels can provide valuable guidance.

### 4.2. Study Limitations

The limitations of this review include the small number of studies available, which limits the generalizability of the findings regarding QES for allergic rhinitis. Additionally, the included studies varied in design, population characteristics, and outcome measures, making it challenging to draw definitive conclusions. Furthermore, potential publication bias cannot be excluded, as studies with negative results may not have been reported.

## 5. Conclusions

In summary, QES combined with zinc can alleviate symptoms of allergic rhinitis, making them a safe and promising integrative option for individuals with this condition, including those using corticosteroids and antihistamine drugs. However, QES should be avoided in patients with egg allergies due to the risk of adverse reactions.

Larger placebo-controlled trials involving more patients with different allergic diseases are needed to confirm these findings. In particular, it would be useful to assess the efficacy of quail egg therapeutic derivatives both with and without zinc supplementation to precisely assess any specific effects of these products, regardless of other compounds associated.

## Figures and Tables

**Table 1:** Nutritional composition per 100 g of raw quail eggs in comparison to other egg varieties.

Nutritional Content		Values for 100 g of Eggs			
		Quail	Chicken	Duck	Goose
Energy		158 kcal	143 kcal	185 kcal	185 kcal
Water		74.4 g	76.2 g	70.8 g	70.4 g
Macronutrients	Protein	13 g	12.6 g	12.8 g	13.9 g
	Total sugars	0.4 g	0.4 g	0.93 g	0.9 g
	Total lipid (fat)	11.10 g	9.51 g	13.80 g	13.30 g
Lipid content	Fatty acids, total saturated	3.56 g	3.13 g	3.68 g	3.60 g
	Fatty acids, total monounsaturated	4.32 g	3.66 g	6.52 g	5.75 g
	Fatty acids, total polyunsaturated	1.32 g	1.91 g	1.22 g	1.67 g
	Cholesterol	844 mg	372 mg	884 mg	852 mg
Relevant micronutrients	Calcium	64 mg	56 mg	64 mg	60 mg
	Iron	3.65 mg	1.75 mg	3.85 mg	3.64 mg
	Magnesium	13 mg	12 mg	17 mg	16 mg
	Phosphorus	226 mg	198 mg	220 mg	208 mg
	Zinc	1.47 mg	1.29 mg	1.41 mg	1.33 mg
	Selenium	32.0 µg	30.7 µg	36.4 µg	36.9 µg
	Vitamin A	156 µg	160 µg	194 µg	187 µg
	Vitamin D	55 IU	82 IU	69 IU	66 IU
	Vitamin E	1.08 mg	1.05 mg	1.34 mg	1.29 mg
	Lutein + zeaxanthin	369 µg	503 µg	459 µg	442 µg

Data extracted from “FoodData Central” of the US Department of Agriculture (link: https://fdc.nal.usda.gov/food-details/172191/nutrients—accessed on 24 January 2025). The average weight of a quail egg is approximately 11.8 g: of this total, the egg’s contents make up nearly 85% (about 10.0 g), while the eggshell accounts for the remaining 15%, weighing approximately 1.77 g [21]. Thus, 100 g of the product is equivalent to around 8 to 10 quail eggs.

**Table 2:** Summary of included studies on the effects of quail egg-based supplements for treating seasonal respiratory allergies.

Population	Intervention (n)	Comparison (n)	Outcomes (Mean ± SD)	Study Design	Citation
690 patients (ITT) with respiratory allergies and IgE levels of at least 300 UI/mL Mean age: ? Gender ratio: ? Study 1 (n = 180—PP): pediatric patients with asthma Studies 2–4 (n = 435—PP: 95/180/160): patients with seasonal AR Study 5 (n = 41—PP): patients with perennial AR and hypersensitivity to indoor allergens	QE + zinc—Ovix^®^ (n = 296) in adjunct to standard therapy Studies 1–4: 1 tablet a day for 75–450 days Study 5: 2 tablets a day for 42 days	Placebo tablets in adjunct to standard therapy (n = 394)	Study 1: 77.7% reduction in asthmatic crises and a 58.6% decrease in the use of rescue medications (*) Studies 2–4: significant reduction in rhinitis severity, improvement in mucosal condition, and decrease in medicinal drug consumption (*) Physician’s evaluation of efficacy: positive outcomes in 76% of pediatric patients and 61% of adult patients (int) vs. 12% of pediatric patients and 13% of adult patients (con) (*) Study 5: Physician’s evaluation of efficacy: positive outcomes in 63% (int) vs. 27% (con) of patients (*)	5 RCTs	[23]
43 atopic subjects Mean age: 32.0 [20; 58] M: 52%; F: 48%	QES + zinc (2 tablets after the allergenic challenge)—SniZtop^®^ (n = 43)	Placebo tablets (n = 43)	15 min after the challenge: PNIF (L/min): −12.79 ± 17.19 vs. −18.75 ± 13.02 (*) VAS (nasal obstruction): 17.07 ± 16.18 vs. 20.78 ± 19.61 (*) Total IgE (IU/mL): 224.68 ± 464.643 vs. 307.14 ± 490.131 (ns)	Crossover RCT (WP: 1 week)	[24]
40 patients with mild-to-severe AR Mean age: 38.5 [19; 51] M: 60%; F: 40%	QES + zinc (1 tablet twice a day for 4 weeks)—Narivent^®^ in adjunct to MNS (n = 20)	MNS every day for 4 weeks (n = 20)	TNSS: 2.95 ± 1.73 (con) vs. 4.5 ± 2.44 (int) (*) Pre-post changes in RCAT (median and IQR): +2 [0; +3.5] (con) vs. +5 [+1.5; +7] (int) (*) Pre-post changes in total IgE (IU/mL): No significant difference between groups	RCT	[25]
77 patients with mild AR Mean age: 41.4 [18; 63] M: 57%; F: 43%	QES + zinc (2 tablets when needed, up to 6 tablets/day for 7 days)—SniZtop^®^ (n = 77)	None (n = 0)	1 day (0–120 min): PNIF (L/min): from 83.0 ± 32.7 to 94.8 ± 32.9 (*) VAS (rhinorrhea): from 18.6 [14.0; 24.6] to 6.9 [4.8; 9.9] 1 week (0–120 min): PNIF (L/min): from 97.0 ± 30.7 to 105.1 ± 32.7 (*) VAS (rhinorrhea): ? (*)	Pre-post study	[26]
45 patients with moderate-to-severe AR Mean age: 32.5 [18; 60] M: 47%; F: 53%	QES + zinc (1 tablet twice a day for 30 days)—Narivent^®^ (n = 45)	None (n = 0)	30 days: SNOT-20: from 38.14 [12; 72] to 26.14 [8; 44] (*) MTT (min): from 24 [19; 40] to 12 [?; ?] (*)	Pre-post study	[27]

F = Females. ITT = Intention-To-Treat (number of patients originally intended to be treated). M = Males. MNS = Mometasone Nasal Spray. MTT = Mucociliary Transport Time. PNIF = Peak Nasal Inspiratory Flow. PP = Per Protocol (number of patients who were actually treated after accounting for dropouts, exclusions, and withdrawals). QES = quail egg-based dietary supplement. RCAT = Rhinitis Control Assessment Test. RCT = Randomized Controlled Trial. SNOT-20 = Sino-Nasal Outcome Test score (20 items). TNSS = Total Nasal Symptom Score. VAS = Visual Analogue Scale. WP = Washout Period. (*) = significant difference in favor of intervention (*p* < 0.05). ? = numerical values not available.

**Table 3:** Quality assessment of the included studies.

First Author (Date)	Design	1	2	3	4	5	6	7	8	9	10	11	12	13	14	Citation
Bruttmann (1995)	RCT	Y	?	Y	Y	?	Y	Y	Y	Y	?	Y	N	?	?	[23]
Benichou (2014)		Y	Y	Y	Y	Y	Y	Y	Y	Y	?	Y	Y	Y	Y	[24]
Andaloro (2022)		Y	Y	Y	Y	Y	Y	Y	Y	Y	Y	Y	N	Y	Y	[25]
Syrigou (2021)	Pre-post study	Y	Y	Y	?	Y	Y	Y	N	Y	Y	Y	Y	/	/	[26]
Passali (2020)		Y	Y	Y	?	?	Y	Y	N	Y	Y	N	Y	/	/	[27]

Y = Yes; N = No; ? = Unclear; / = Not applicable. For a full description of each item, refer to the NIH study quality assessment tool, accessible online at https://www.nhlbi.nih.gov/health-topics/study-quality-assessment-tools (date of consultation: 23 January 2025).

**Table 4:** Adverse effects reported in studies investigating the efficacy of QES + zinc for allergic rhinitis.

First Author (Date)	Adverse Effects Reported in Each Study	Citation
Bruttmann (1995)	No clinically relevant adverse effects	[23]
Benichou (2014)	No adverse effects	[24]
Andaloro (2022)	Adverse effects were reported in two patients (10%) for each group: in the intervention group, two patients had nasal dryness; in the control group, one patient developed nasal dryness and another one had epistaxis	[25]
Syrigou (2021)	Two patients (2.6%) reported muscle strain and cough	[26]
Passali (2020)	No clinically relevant adverse effects	[27]

## Data Availability

All data are available by contacting the corresponding author.
